# Immigration and Gender as Social Determinants of Mental Health during the COVID-19 Outbreak: The Case of US Latina/os

**DOI:** 10.3390/ijerph18116065

**Published:** 2021-06-04

**Authors:** Barbara Gomez-Aguinaga, Melanie Sayuri Dominguez, Sylvia Manzano

**Affiliations:** 1 School of Public Administration, University of Nebraska at Omaha, Omaha, NE 68182, USA; 2 Department of Political Science, University of New Mexico, Albuquerque, NM 87131, USA; msonntag@unm.edu; 3 BSP Research, Agoura Hills, CA 91301, USA; sylvia.manzano@latinodecisions.com

**Keywords:** COVID-19, health inequities, mental health, pandemic, intersectionality, Latina/os, immigration, social determinants of health

## Abstract

While men and women make up a similar number of COVID-19 cases, and are equally likely to know someone who has become ill due to the virus, the gendered and systemic implications of immigration during public health emergencies among minority groups in the United States are empirically underexplored. Using the SOMOS COVID-19 Crisis National Latino Survey, we conduct a series of intersectional analyses to understand the extent to which personal experiences with COVID-19, gendered structural factors, and spillover effects of US immigration policies impact the mental health of US Latina/os during a public health emergency. The results show that among Latinas, knowing an undocumented immigrant and someone ill with COVID-19 increases the probability of reporting worse mental outcomes by 52 percent. Furthermore, being a woman increases the probability of reporting the highest level of mental health problems by 30 percent among Hispanic people who know someone with COVID-19 and an undocumented immigrant. These findings indicate that the effects of the COVID-19 outbreak among US Latinas and Latinos are entrenched in gendered and systemic inequalities.

## 1. Introduction

As the coronavirus pandemic continues to claim the lives of hundreds of thousands of people around the globe, much attention has focused on the United States. With over 33 million infections and 500,000 deaths as of May 2021 [1] that disproportionately affect racial and ethnic minorities (JHU 2021), the COVID-19 pandemic accentuates the racialized, gendered, and political inequalities existing in the country. U.S. data shows that despite being more likely to take the necessary precautions to reduce the spread of the virus, racial and ethnic minorities are more likely to become infected and die with the virus than non-Hispanic white people, due to structural and institutional inequalities [2,3]. People of color and women make up the majority of essential workers who are at increased risk of contracting the virus, due to ongoing exposure to other individuals and patients with COVID-19 [4]. Furthermore, despite their vast presence in the healthcare industry, women are left out from leadership positions and decision-making groups, preventing the spread of COVID-19 [5]. These inequalities require analyses that incorporate the intersectional and preexisting conditions exacerbating the impact of this public health crisis among underrepresented groups.

With ethno-racial minorities being disproportionately affected by COVID-19 infections and deaths, and the unique challenges that women face during public health emergencies—such as disproportionate economic impacts, and caregiving for the sick and out-of-school children [6]—we examine the intersection of gender and connections to immigration as social determinants of mental health of US Latina/os during the COVID-19 outbreak. Using the SOMOS COVID-19 Crisis National Latino Survey [7], we investigate how Latinas’ personal experiences with COVID-19 and their systemic marginalization, due to their gender and connections to immigrants impact their mental health outcomes. The results show that while Latinos and Latinas are equally likely to know someone with COVID-19, the adverse implications of the pandemic on mental health are more pronounced among Latinas, particularly those who have personal connections with undocumented immigrants.

### 1.1. Mental Health Outcomes: The Intangible Implications of Public Health Emergencies

Although the primary focus of the government during public health emergencies is to ensure the physical safety of people within its borders, many have noted that the COVID-19 pandemic has led to a mental health crisis with anxiety, social isolation, and economic stress exacerbating the existing problem of high suicide rates [8,9] and other mental health problems [10,11,12]. Mental health research into past outbreaks, such as Ebola and the Severe Acute Respiratory Syndrome (SARS) have shown that not only survivors, but also family members, healthcare workers, and even the general public suffered from Post-traumatic Stress Disorder (PTSD), anxiety, and depression [13,14,15]. As this research demonstrates, public health emergencies exacerbate existing health inequities that require attention, including access to health insurance, medical care, language barriers, and legal mechanisms that disproportionately penalize immigrants and communities of color [16,17,18]. Considering that mental health issues are connected and influenced by multiple factors in one’s life, we argue that it is important to understand the connection between mental health issues and other systemic inequalities to get a sense of how public health emergencies adversely affect people’s mental health. We focus specifically on the case of US Latinas and Latinos, and examine how multiple layers of inequalities exacerbate and worsen these mental health outcomes.

Public health emergencies come with a number of other consequences not directly related to the disease itself, many of which disproportionately impact minority groups and communities of color in the United States. In the case of COVID-19, another prominent factor is the economic fallout that has resulted in uncertainty and loss of employment for millions of people; this type of economic uncertainty can increase suicide, depression, and anxiety disorders [19,20]. American Indians, African Americans, and Latina/os have the highest percentages of communities living below poverty and are struggling the most during this pandemic [21]. Furthermore, studies have shown that essential workers, many of whom are racial and ethnic minorities, can face aggravating mental health challenges during public health emergencies [10,22]. Hence, it is evident that public health emergencies, including the COVID-19 outbreak, exacerbate existing inequalities and hit those at the margins the hardest.

### 1.2. An Intersectional Approach on Mental Health Outcomes during COVID-19

How do gendered and systemic factors affect the mental health of US Latina/os during the COVID-19 pandemic? To analyze this question, we use an intersectional approach that highlights the unique and longstanding challenges that Latina/os have experienced in the United States, focusing on gender and their connections to immigration [23,24]. Intersectionality is a paradigm that helps us understand the diversity and complexity of lives within groups, and their implications on multiple outcomes, including the physical and mental health of minority groups [25,26]. For example, regardless of their socioeconomic status, Black women are almost three times more likely to die from pregnancy-related causes than white women [27,28]. Similarly, women are over two times more likely to experience depression and anxiety than men [29], and African American women are less likely than white women to seek medical help, due to structural barriers, such as discrimination and lack of health insurance [30]. Along with a well-established body of literature, Eaton and colleagues [29] attribute this to the difference in how men and women process emotions. Reports have highlighted that stigma around mental health prevents many men from seeking help, which could be attributed to gender norms and differences in how men and women are socialized [31], whereas others have argued that these differences are biologically driven. While this debate in the literature is immense, we focus on the outcomes—rather than the outputs—of health differences among men and women. Hence, the use of intersectionality to analyze the mental health of women of color is crucial, particularly during public health emergencies that exacerbate recurring inequalities [32,33].

#### 1.2.1. Gendered Health

The quality and access to health care in the United States has been declining over many years. With high health care costs and access being linked to employment status, and not all employers providing health care for their workers, vulnerable and marginalized populations are most affected [16]. Furthermore, social movements and multiple studies have highlighted the gendered nature of the health care system in the United States for decades. Reproductive care and the politicization of reproductive rights, the lack of paid maternity leave, access to and treatment in health care, and biases in medical research exemplifying these inequalities [34,35,36,37].

Women of color experience the gendered health system not only through their gender but also their race or ethnicity. The intersectional experience of US Latina/os is, therefore, both gendered and racialized. In recent years, Latinas have emerged as a “powerful force in the U.S. job market”, with the highest labor participation rates among women, due to raising education attainment [38]. However, most Latina/o immigrants arriving in the U.S. are now women, due to gendered employment opportunities and human rights abuses targeting women in their home countries [39]. Additionally, Latinas continuously face stressors that are proven to impact their physical and mental health, more so than their male counterparts, such as lack of access to health insurance and health care services [40], as well as oppressive experiences (e.g., sexism, racism, sexual objectification), and trauma [41,42].

#### 1.2.2. Immigration as a Social Determinant of Health

Racial and ethnic inequalities in health outcomes are abundant in the United States—ranging from physical health, such as cardiovascular diseases and asthma, to mental health outcomes, such as PTSD and suicide [43,44]. These disparities exist due to systemic inequalities, such as economic opportunities, segregation, educational attainment, and access to health care [45,46]. However, some minority groups, such as US Latina/os, face additional burdens that become determinants of health, including language barriers, immigration status, and connections to immigrants [17,44,47].

The spillover effects of immigration policies and practices in the United States are among the most notorious and recently acknowledged social determinants of health among Latina/os. Studies have shown that immigration enforcement in the United States is inhumane and racialized, disproportionately targeting immigrants of color—most of them from Latin America [18,48,49]. Moreover, the criminalization of immigration and the dehumanizing conditions in detention centers have detrimental effects on the well-being and mental health of Latina/o immigrants and their communities, including US-born adults and children. Studies have found that, due to increasing immigration surveillance at all levels of government, having personal connections with immigrants disrupts the daily lives of Latina/os by avoiding contact with police, education services, and health care providers [18,50]. Latina/o adults who have connections with undocumented immigrants or deportees are more likely to require medical help for mental health problems, and their children are more likely to be diagnosed with developmental disorders [18,51]. Immigrant experiences among Latina/os serve as social determinants of mental health because social locations, traumatic experiences, and the unequal distribution of resources and opportunities serve as sources of stress and inequalities. These disruptions, at the same time, impact the individuals experiencing such difficulties and those who surround them, as the Stress Process Theory posits [52,53]. Hence, the challenges and inequalities that the foreign-born population faces in the United States lead to negative health outcomes for immigrants themselves, as well as those who surround them, including US-born families, friends, and community members.

#### 1.2.3. Intersectionality and COVID-19

During public health emergencies and disasters, vulnerable communities are at increased risk of suffering the most severe consequences. Racial and ethnic minorities have been found to be particularly vulnerable—both physically and psychologically [54]. Coupled with the extensive research that finds poorer health outcomes for ethno-racial minorities that can be attributed to structural discrimination, public health emergencies present a dire situation for already vulnerable populations [55,56]. In the current pandemic, government responses and resources have primarily addressed physical health during public health emergencies. Although this course of action is sound to contain the disease and treat those affected, it is also crucial to address the mental health impacts that public health emergencies have on a surviving population. While studies have found that past public health emergencies have unequal effects on marginalized communities [13,15], the mental health inequalities that emerge from public health emergencies among women of color are empirically underexplored. Hence, incorporating an intersectional analysis during the current pandemic is highly important among Latina/os, who face multiple experiences affecting their mental health, gendered structures, and connections to immigration. Based on existing literature on public health emergencies, social determinants of health, and intersectionality, we expect that among Latina/os, those who have a connection with an undocumented person, identify as women, and know someone ill with COVID-19 experience the most adverse mental health outcomes during the current pandemic.

## 2. Materials and Methods

**Figure 1:**
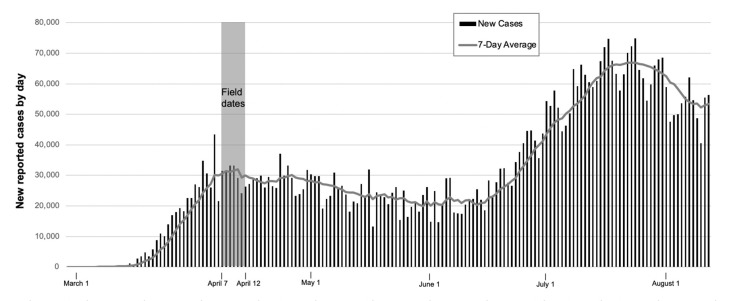
Daily reported COVID-19 cases in the United States. Source: Centers for Disease Control and Prevention, “New Cases by Day”.

To test the extent to which gender and connections to immigrants are social determinants of mental health during the COVID-19 outbreak among US Latinas and Latinos, we use the SOMOS COVID-19 Crisis National Latino Survey (n = 1200), which has a nationally representative sample of US Latina/os; although this study is based on Latina/os who self-identified as “female” or “male”, we acknowledge the growing presence of LGBTQI+ Latina/os in the United States [57]. The survey was conducted between 7–12 April 2020, when over 189,000 new coronavirus cases and more than 12,000 deaths were reported [7,58]. This was the first and most comprehensive survey of Latina/os on the impact and reactions to COVID-19 and was conducted during the first national peak for COVID-19 cases, as Figure 1 shows.

The main dependent variable of the study is reported mental health, which consists of a Likert scale obtained from the following questions: “Over the last 2 weeks, how often have you been experiencing any of the following: (1) Feeling nervous, anxious or on edge; (2) Not being able to stop or control worrying; (3) Little interest or pleasure in doing things; and (4) Feeling down, depressed, or hopeless” [59]. Hence, the reported mental health variable ranges from 0—meaning no mental health problems, to 4—experiencing all of the mental health issues.

This study includes three main independent variables: (1) Personal experiences with COVID-19, which was obtained by asking survey respondents, “Do you have any friends or family who have become ill due to the coronavirus?” Yes/No; (2) knowing an undocumented immigrant, obtained by asking, “Thinking about the people in your family as well as your friends and co-workers, do you happen to know someone who is an undocumented immigrant?” Yes/No; and (3) gender, coded as female or male. We include various covariates to account for the contexts and demographic characteristics of survey respondents, such as nativity, education, age, marital status, income, Mexican ancestry, language of the survey, and access to health insurance, as previous studies on the subject have shown their implications on health outcomes among minority groups [17,18]. Table 1 presents the summary statistics of the variables used in this study.

We conduct a series of ordered logistic regressions in Stata/MP 16 to account for the extent to which personal experiences with COVID-19, gendered structural factors, and connection to immigrants affect the mental health of US Latinas during a public health emergency. The first model is an ordered logistic regression with reported mental health as the dependent variable, and gender, personal experiences with COVID-19, and knowing an undocumented immigrant as the main independent variables. The second model presents an ordered logistic regression with a three-way interaction among gender, knowing someone with COVID-19, and knowing an undocumented immigrant to analyze their intersectional implications on the mental health of US Latina/os during the beginning of the COVID-19 outbreak. Appendix A presents the model specification of the ordered logit regressions of this study.

## 3. Results

Table 2 presents the results of the order logistic regression with reported mental health as the dependent variable, in which higher levels of mental health indicate experiencing more mental health problems. The odds ratios reveal that women are significantly more likely (48.6%) to report higher levels of mental health issues than their men counterparts, whereas knowing someone with COVID-19 is marginally associated with higher mental health problems (25%) compared to those who do not know someone potentially ill with COVID-19. These findings provide support for the gendered implications of public health emergencies on the mental health of women and call for additional exploration on the role of personal experiences with illnesses during pandemics [60,61].

One of the most salient findings of Table 2 relates to the structural factors of nativity. The results show that while nativity per se is not significantly associated with reporting mental health problems among US Latina/os, knowing an undocumented immigrant significantly increases the odds of reporting higher levels of mental health problems by 67.9%. While this finding seems irreconcilable, it is in line with literature on public health asserting that connections to immigrants, particularly knowing a deportee or an undocumented immigrant, have adverse implications on the health and well-being of American citizens [62,63].

**Figure 2:**
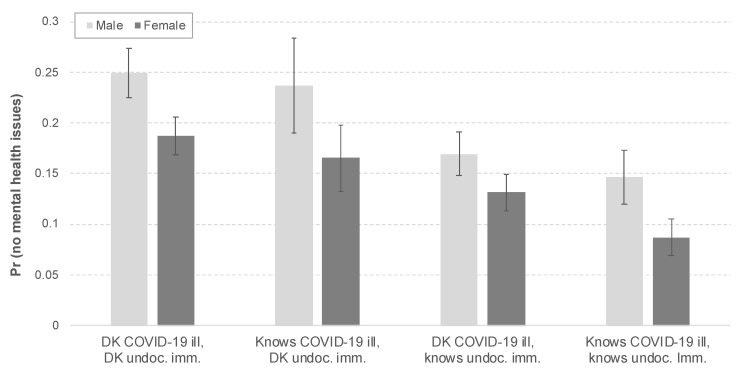
Predicted probabilities of reporting no mental health issues (three-way interaction among gender, knowing someone with COVID-19, and knowing undocumented immigrant). Source: Authors’ analysis using the SOMOS COVID-19 Crisis National Latino Survey (2020).

Table 3 presents the results of the ordered logistic regression with reported mental health as the dependent variable, with a three-way interaction among gender, knowing someone ill with COVID-19, and knowing an undocumented immigrant. Although the coefficients of the interactions show no statistical significance, the predicted probabilities of the three-way interaction reveal that the mental health implications of the current pandemic among Latina/os are intersectional; Appendix B presents the predicted probabilities of the three-way interaction from the order logistic regression analysis. Figure 2 presents the predicted probabilities of reporting no mental health problems by groups. Overall, the results show that Latino men are more likely to report no mental health problems compared to Latinas. However, the results also vary across lived experiences. Across genders, Latino men who do not know either someone ill with COVID-19 or an undocumented immigrant are the most likely to report no mental health issues, whereas Latinas who know someone ill with COVID-19 and an undocumented immigrant are the least likely to report no mental health problems. Among men, Figure 2 shows that the effect of knowing an undocumented immigrant and someone with COVID-19 reduces the predicted probability of reporting no mental health problems by 41 percent (corresponding with the 10-percentage point difference). In other words, the predicted probabilities show that gendered and structural barriers decrease the likelihood of not having mental health problems among US Latina/os.

**Figure 3:**
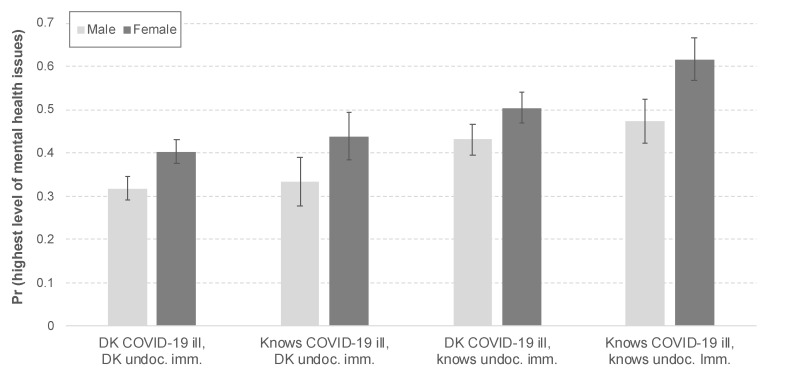
Predicted probabilities of reporting the highest level of mental health issues (three-way interaction among gender, knowing someone with COVID-19, and knowing undocumented immigrant). Source: Authors’ analysis using the SOMOS COVID-19 Crisis National Latino Survey (2020).

Figure 3 presents the predicted probabilities of reporting the highest level of mental health problems by the group. Concurring with the argument advanced in Figure 2, the results show that while women at large are more likely to report the highest level of mental health problems than men, the detrimental implications of the pandemic are intersectional. Figure 3 shows that the intersectional effects of knowing someone ill with COVID-19, and knowing an undocumented immigrant increase the predicted probability of reporting the highest level of mental problems by 52 percent (corresponding with a difference of 21.3 percentage points) among women. Similarly, among respondents who know someone with COVID-19, as well as an undocumented immigrant, the effect of being a woman increases the predicted probability of reporting the highest level of mental health problems by 30 percent (corresponding with a 14.3 percentage point difference) compared to men. These findings provide evidence of the multiple challenges and oppressions that Latinas face not only as gender minorities, but also through their connections to the immigrant experience in the United States. These results show that gender and the spillover effects of immigration policies are social determinants of mental health among US Latina/os.

## 4. Discussion

Our research examines the intersectional implications of the COVID-19 pandemic on the mental health of US Latina/os, finding that the effects are not only gendered but also contingent on systemic inequalities that revolve around the spillover effects of immigration policies. The results show that connections to undocumented immigrants aggravate the mental health outcomes of US Latina/os during the current pandemic. Individual experiences (identifying as female, knowing someone ill with COVID-19, and knowing an undocumented immigrant) have an impact on the adverse effects of the mental health of US Latina/os; these findings concur with existing literature on social determinants of health [55,61]. More importantly, this study increases our understanding of the intersectional implications of the lived experiences of US Latinos and Latinas, who have disproportionately faced adverse effects during the COVID-19 pandemic.

We illustrate that during the COVID-19 outbreak, Latinas’ personal connections to the US immigration system and the COVID-19 pandemic have had significant detrimental implications on their mental health, more so than their Latino male counterparts. Among Latinas, knowing someone ill with COVID-19, and knowing an undocumented immigrant increase the probability of reporting worse mental outcomes by 52 percent. Additionally, being a woman increases the probability of reporting the highest level of mental health problems by 30 percent among Latina/o respondents who know someone with COVID-19, as well as an undocumented immigrant, compared to men. This finding highlights the importance of intersectional analyses in health outcomes of communities of color, who will soon make up the majority of the country [64].

While intersectionality studies present methodological challenges to account for multiple identities across marginalized groups, we urge scholars to continue to examine social determinants of health, particularly during public health emergencies, through intersectional approaches. Besides theoretical and qualitative analyses [65,66], larger studies have raised awareness of the “potential and promise that intersectionality holds as a lens for studying the social determinants of health, reducing health disparities, and promoting health equity” [60]. Social and health sciences have increasingly considered factors outside of genetics to understand the extent to which social and political environments impact health interventions and outcomes [22,66]. However, the incorporation of research projects centered on intersectionality has the potential to not only create innovative research but also lead to practical public policies and interventions to advance health and well-being outcomes among disadvantaged communities [60]. Health equity, as the COVID-19 pandemic has stressed, is a crucial area of study in rapidly changing and growingly diverse democracies, such as the United States.

## 5. Conclusions

In this paper, we investigated the relationship between immigration and gender as social determinants of health by looking at mental health outcomes for US Latina/os during the COVID-19 pandemic. Using the SOMOS COVID-19 Crisis National Latino Survey, we conducted ordered logistic regressions, and found that for Latinas, knowing an undocumented immigrant and someone ill with COVID-19 increased the probability of reporting worse mental health outcomes by 52 percent. Our research showed that while gender is a crucial predictor for self-reported mental health, with Latinas being more likely to report mental health problems compared to Latinos, lived experiences, including knowing an undocumented immigrant and knowing someone ill with COVID, also affect mental health outcomes.

## 6. Recommendations and Avenues for Future Research

Our study contributes to our understanding of the social determinants of mental health during the COVID-19 pandemic among the largest ethnic group in the United States through an intersectional approach. However, there are limitations related to the nature of the data that future scholarship shall address. Given that we rely on cross-sectional data that is centered on Latina/o populations, the data limits our ability to make generalizations across racial and ethnic groups other than Latina/os over time. Moreover, the data of this study were collected during the COVID-19 outbreak, a period of time that may be socially and/or politically distinct to other pandemics and public health emergencies. With those limitations in mind, we encourage scholars to continue to analyze the effects of intersectionality on public health and health outcomes, particularly across racial and ethnic groups over time.

## Figures and Tables

**Table 1:** Summary statistics of the variables used in this study.

Variable	Observations	Mean	Std. Dev.	Min–Max
Mental health	1202	2.433	1.581	0–4
Female	1200	0.520	0.500	0–1
Knows COVID-19 ill	1200	0.253	0.435	0–1
Knows undocumented immigrant	1200	0.462	0.499	0–1
US-born	1200	0.600	0.490	0–1
Education	1200	3.003	1.539	1–6
Age	1083	2.222	1.012	1–4
Married	1200	0.428	0.495	0–1
Income	1196	2.834	1.803	1–7
Uninsured	1200	0.716	0.451	0–1
Mexican ancestry	1202	0.408	0.492	0–1
Spanish survey	1200	0.414	0.493	0–1

Source: SOMOS COVID-19 Crisis National Latino Survey (2020).

**Table 2:** Effect of gender and personal experiences on mental health during COVID-19 outbreak.

	β	Odds
Variables	(Standard Errors)	Ratio
Female	0.396 **	1.486 **
	(0.114)	
Knows COVID-19 ill	0.224 *	1.250 *
	(0.135)	
Knows undocumented immigrant	0.518 **	1.679 **
	(0.118)	
US-born	0.229	1.257
	(0.142)	
Education	0.099 **	1.104 **
	(0.038)	
Age category	−0.197 **	0.821 **
	(0.061)	
Married	0.037	1.038
	(0.124)	
Income	−0.095 **	0.910 **
	(0.034)	
Uninsured	-0.151	0.860
	(0.129)	
Mexican ancestry	−0.059	0.943
	(0.119)	
Spanish survey	−0.251 *	0.778 *
	(0.143)	
Observations	1079	1079

Source: Authors’ analysis using the SOMOS COVID-19 Crisis National Latino Survey (2020). ** *p* < 0.05, * *p* < 0.1.

**Table 3:** Ordered logistic regression assessing the intersectional implications of COVID-19 on mental health (three-way Interaction among gender, knowing someone with COVID-19, and knowing an undocumented immigrant).

	β	Odds
Variables	(Standard Errors)	Ratio
Female	0.381 **	1.464 **
	(0.168)	
Knows COVID-19 ill	0.074	1.077
	(0.287)	
Knows undocumented immigrant	0.503 ***	1.653 ***
	(0.193)	
Female x Knows COVID-19 ill x	0.219	1.244
Knows undocumented immigrant	(0.531)	
Female x Knows COVID-19 ill	0.077	1.081
	(0.386)	
Female x Knows undocumented	−0.077	0.926
immigrant	(0.265)	
Knows COVID-19 ill x Knows	0.103	1.108
undocumented immigrant	(0.383)	
US-born	0.234 *	1.264 *
	(0.142)	
Education	0.100 ***	1.105 ***
	(0.038)	
Age category	−0.200 ***	0.819 ***
	(0.062)	
Married	0.041	1.042
	(0.124)	
Income	−0.097 ***	0.908 ***
	(0.034)	
Uninsured	−0.154	0.857
	(0.129)	
Mexican ancestry	−0.054	0.947
	(0.119)	
Spanish survey	−0.246 *	0.782 *
	(0.143)	
Observations	1079	1079

Source: Authors’ analysis using the SOMOS COVID-19 Crisis National Latino Survey (2020). *** *p* < 0.01, ** *p* < 0.05, * *p* < 0.1.

## Data Availability

The data that supports the findings of this study belongs to Latino Decisions and BSP Research, and may be available upon request from these organizations. The complete instrument and survey results are available at LatinoDecisions.com.

## Appendix A. Model Specification

The following ordered logit model was used for the estimation conducted in Stata/MP 16:

$$Y_{i}^{*}=\overset{k}{\underset{i=1}{\sum }}\beta_{k}X_{ki}+\varepsilon_{i}=Z_{i}+\varepsilon_{i}$$

where X is the vector of the covariates capturing the respondents’ gender (female), knowing someone with COVID-19, nativity (US-born), education, age, marital status (married), income, access to health insurance (uninsured), Mexican ancestry, and language of the survey (Spanish survey). The estimated value of Z and the residual term can predict the probability that the unobserved variable Y^*^ is located within the threshold limits of the dependent variable of the study [67,68].

## Appendix B. Predicted Probabilities of the Three-Way Interaction

**Table A1:** Predicted probabilities of the three-way interaction among gender, knowing someone with COVID-19, and knowing an undocumented immigrant.

Mental Health Issues	Characteristics	Margin	SE	CI
0	Male, DK COVID-19 ill, DK undoc. imm.	0.250	0.024	0.202–0.297
	Male, DK COVID-19 ill, knows undoc. imm.	0.170	0.022	0.127–0.212
	Male, knows COVID-19 ill, DK undoc. imm.	0.236	0.047	0.145–0.328
	Male, knows COVID-19 ill, knows undoc. imm.	0.147	0.027	0.094–0.199
	Female, DK COVID-19 ill, DK undoc. imm.	0.187	0.019	0.150–0.224
	Female, DK COVID-19 ill, knows undoc. imm.	0.132	0.018	0.096–0.167
	Female, knows COVID-19 ill, DK undoc. imm.	0.165	0.033	0.102–0.229
	Female, knows COVID-19 ill, knows undoc. imm.	0.087	0.018	0.052–0.122
1	Male, DK COVID-19 ill, DK undoc. imm.	0.127	0.012	0.103–0.151
	Male, DK COVID-19 ill, knows undoc. imm.	0.101	0.012	0.078–0.124
	Male, knows COVID-19 ill, DK undoc. imm.	0.123	0.016	0.091–0.155
	Male, knows COVID-19 ill, knows undoc. imm.	0.092	0.014	0.065–0.119
	Female, DK COVID-19 ill, DK undoc. imm.	0.108	0.011	0.086–0.129
	Female, DK COVID-19 ill, knows undoc. imm.	0.085	0.011	0.064–0.106
	Female, knows COVID-19 ill, DK undoc. imm.	0.100	0.015	0.070–0.129
	Female, knows COVID-19 ill, knows undoc. imm.	0.062	0.011	0.040–0.084
2	Male, DK COVID-19 ill, DK undoc. imm.	0.139	0.011	0.117–0.161
	Male, DK COVID-19 ill, knows undoc. imm.	0.125	0.012	0.102–0.147
	Male, knows COVID-19 ill, DK undoc. imm.	0.138	0.012	0.114–0.161
	Male, knows COVID-19 ill, knows undoc. imm.	0.117	0.013	0.092–0.143
	Female, DK COVID-19 ill, DK undoc. imm.	0.129	0.011	0.108–0.151
	Female, DK COVID-19 ill, knows undoc. imm.	0.111	0.011	0.089–0.133
	Female, knows COVID-19 ill, DK undoc. imm.	0.124	0.013	0.097–0.150
	Female, knows COVID-19 ill, knows undoc. imm.	0.088	0.013	0.062–0.113
3	Male, DK COVID-19 ill, DK undoc. imm.	0.167	0.012	0.144–0.190
	Male, DK COVID-19 ill, knows undoc. imm.	0.173	0.012	0.150–0.196
	Male, knows COVID-19 ill, DK undoc. imm.	0.169	0.013	0.143–0.195
	Male, knows COVID-19 ill, knows undoc. imm.	0.171	0.012	0.147–0.195
	Female, DK COVID-19 ill, DK undoc. imm.	0.173	0.012	0.150–0.196
	Female, DK COVID-19 ill, knows undoc. imm.	0.168	0.012	0.144–0.191
	Female, knows COVID-19 ill, DK undoc. imm.	0.173	0.012	0.150–0.196
	Female, knows COVID-19 ill, knows undoc. imm.	0.148	0.015	0.119–0.177
4	Male, DK COVID-19 ill, DK undoc. imm.	0.318	0.027	0.265–0.371
	Male, DK COVID-19 ill, knows undoc. imm.	0.431	0.036	0.362–0.501
	Male, knows COVID-19 ill, DK undoc. imm.	0.334	0.057	0.223–0.445
	Male, knows COVID-19 ill, knows undoc. imm.	0.473	0.051	0.374–0.573
	Female, DK COVID-19 ill, DK undoc. imm.	0.403	0.028	0.348–0.457
	Female, DK COVID-19 ill, knows undoc. imm.	0.504	0.036	0.433–0.575
	Female, knows COVID-19 ill, DK undoc. imm.	0.438	0.056	0.329–0.547
	Female, knows COVID-19 ill, knows undoc. imm.	0.616	0.050	0.519–0.713

Source: Authors’ analysis from ordered logistic regression with three-way interaction among gender, knowing someone with COVID-19, and knowing an undocumented immigrant, using the SOMOS COVID-19 Crisis National Latino Survey (2020).

## References

[1] John Hopkins University (JHU) COVID-19 Dashboard by the Center Systems Science and Engineering (CSSE). https://coronavirus.jhu.edu/map.html.

[2] (2020). Structural Inequalities and Not Behavior Explain Covid-19 Racial Disparities. https://www.latinorebels.com/2020/05/01/structuralinequalitiescovid/..

[3] (2020). Compliant but Unprotected: Communities of Color. Take Greater Action to Prevent the Spread of COVID-19 but Remain at Risk. http://compliant-but-unprotected-communities-of-color-take-greater-action-to-prevent-the-spread-of-covid-19-but-remain-at-risk/.

[4] Powell C. (2020). The Color and Gender of COVID: Essential Workers, Not Disposable People. https://www.thinkglobalhealth.org/article/color-and-gender-covid-essential-workers-not-disposable-people.

[5] (2020). Where the Women Aren’t: On Coronavirus Task Forces. https://www.npr.org/sections/goatsandsoda/2020/06/24/882109538/where-the-women-arent-on-coronavirus-task-forces.

[6] Algasseer N., Dresden E., Keeney G., Warren N. (2004). Status of women and infants in complex humanitarian emergencies. J. Midwif. Women’s Health.

[7] Latino Decisions (2020). SOMOS—COVID-19 Crisis National Latino Survey. https://latinodecisions.com/polls-and-research/somos-covid-19-crisis-national-latino-survey-april-2020/.

[8] Reger M.A., Stanley I.H., Joiner T.E. (2020). Suicide Mortality and Coronavirus Disease 2019—A Perfect Storm?. JAMA Psychiatry.

[9] Sher L. (2020). The impact of the COVID-19 pandemic on suicide rates. QJM Int. J. Med..

[10] Greenberg N., Docherty M., Gnanapragasam S., Wessely S. (2020). Managing mental health challenges faced by healthcare workers during covid-19 pandemic. BMJ.

[11] Hsu C.-H., Lin H.-H., Wang C.-C., Jhang S. (2020). How to Defend COVID-19 in Taiwan? Talk about People’s Disease Awareness, Attitudes, Behaviors and the Impact of Physical and Mental Health. Int. J. Environ. Res. Public Health.

[12] Qiu J., Shen B., Zhao M., Wang Z., Xie B., Xu Y. (2020). A nationwide survey of psychological distress among Chinese people in the COVID-19 epidemic: Implications and policy recommendations. Gen. Psychiatry.

[13] Jalloh M.F., Li W., Bunnell R.E., Ethier K.A., O’Leary A., Hageman K.M., Sengeh P., Jalloh M.B., Morgan O., Hersey S. (2018). Impact of Ebola experiences and risk perceptions on mental health in Sierra Leone, July 2015. BMJ Glob. Health.

[14] Lee S.M., Kang W.S., Cho A.-R., Kim T., Park J.K. (2018). Psychological impact of the 2015 MERS outbreak on hospital workers and quarantined hemodialysis patients. Compr. Psychiatry.

[15] Mak I.W.C., Chu C.M., Pan P.C., Yiu M.G.C., Chan V.L. (2009). Long-term psychiatric morbidities among SARS survivors. Gen. Hosp. Psychiatry.

[16] Case A., Angus D. (2020). Deaths of Despair and the Future of Capitalism.

[17] Sanchez G.R., Vargas E.D., Juarez M.D., Gomez-Aguinaga B., Pedraza F.I. (2017). Nativity and citizenship status affect Latinos’ health insurance coverage under the ACA. J. Ethn. Migr. Stud..

[18] Juárez M., Gomez-Aguinaga B., Bettez S.P. (2018). Twenty years after IIRIRA: The rise of immigrant detention and its effects on Latinx communities across the nation. J. Migrat. Human Securit..

[19] Dijkstra-Kersten S.M.A., Biesheuvel-Leliefeld K.E.M., van der Wouden J., Penninx B.W.J.H., Van Marwijk H.W.J. (2015). Associations of financial strain and income with depressive and anxiety disorders. J. Epidemiol. Community Health.

[20] Reeves A., Stuckler D., McKee M., Gunnell D., Chang S.-S., Basu S. (2012). Increase in state suicide rates in the USA during economic recession. Lancet.

[21] Karpman M., Dulce G., Genevieve M.K. (2020). Parents are Struggling to Provide for Their Families during the Pandemic.

[22] Mwarumba N. (2017). Global Social Vulnerability to Pandemics: An Examination of Social Determinants of H1N1 2009 Mortality. Ph.D. Thesis.

[23] López N., Vargas E.D., Juarez M., Cacari-Stone L., Bettez S. (2018). What’s Your Street Race? Leveraging Multidimensional Measures of Race and Intersectionality for Examining Physical and Mental Health Status among Latinxs. Sociol. Race Ethn..

[24] Sampaio A. (2014). Racing and gendering immigration politics: Analyzing contemporary immigration enforcement using intersectional analysis. Pol. Groups Ident..

[25] Collins P.H. (2002). Black Feminist Thought: Knowledge, Consciousness, and the Politics of Empowerment.

[26] Seng J.S., Lopez W.D., Sperlich M., Hamama L., Meldrum C.D.R. (2012). Marginalized identities, discrimination burden, and mental health: Empirical exploration of an interpersonal-level approach to modeling intersectionality. Soc. Sci. Med..

[27] CDC (Centers for Disease Control and Prevention) Racial and Ethnic Disparities Continue in Pregnancy-Related Deaths. https://www.cdc.gov/media/releases/2019/p0905-racial-ethnic-disparities-pregnancy-deaths.html.

[28] Mays V.M., Cochran S.D., Barnes N.W. (2007). Race, Race-Based Discrimination, and Health Outcomes Among African Americans. Annu. Rev. Psychol..

[29] Eaton N.R., Keyes K.M., Krueger R.F., Balsis S., Skodol A.E., Markon K.E., Grant B.F., Hasin D.S. (2012). An invariant dimensional liability model of gender differences in mental disorder prevalence: Evidence from a national sample. J. Abnorm. Psychol..

[30] Richards E.M. Mental Health Among African-American Women. https://www.hopkinsmedicine.org/health/wellness-and-prevention/mental-health-among-african-american-women.

[31] Campbell L. (2019). Why Many Men Have a Harder Time Seeking Treatment for Mental Illness. https://www.healthline.com/health-news/how-can-we-reduce-mens-mental-health-stigma.

[32] Lawrance B. (2018). Ebola’s Would-be Refugees: Performing Fear and Navigating Asylum During a Public Health Emergency. Med. Anthr..

[33] Petchesky R.P. (2016). Biopolitics at the crossroads of sexuality and disaster: The case of Haiti. The Ashgate Research Companion to the Globalization of Health.

[34] Vedam S., Council G.-U.S., Stoll K., Taiwo T.K., Rubashkin N., Cheyney M., Strauss N., McLemore M., Cadena M., Nethery E. (2019). The Giving Voice to Mothers study: Inequity and mistreatment during pregnancy and childbirth in the United States. Reprod. Health.

[35] Kent J.A., Patel V., Varela N.A. (2012). Gender Disparities in Health Care. Mt. Sinai J. Med. A J. Transl. Pers. Med..

[36] Holdcroft A. (2007). Gender bias in research: How does it affect evidence based medicine?. J. R. Soc. Med..

[37] Thresia C.U., Katia S.M. (2011). Gender bias in health research: Implications for women’s health in Kerala (India) and Sri Lanka. Crit. Public Health.

[38] (2019). Latinas Emerge as a Powerful Force in the U.S. Job Market. Los Angeles Times.

[39] Hallock J., Ariel G.R.S., Michael F. (2018). In Search of Safety, Growing Numbers of Women Flee Central America.

[40] Desai S., Samari G. (2020). COVID-19 and Immigrants’ Access to Sexual and Reproductive Health Services in the United States. Perspect. Sex. Reprod. Health.

[41] Watson L.B., Deblaere C., Langrehr K.J., Zelaya D.G., Flores M.J. (2016). The influence of multiple oppressions on women of color’s experiences with insidious trauma. J. Couns. Psychol..

[42] Berchick E. (2018). Most Uninsured Were Working-Age Adults.

[43] Office of Disease Prevention and Health Promotion (ODPHP) Disparities. https://www.healthypeople.gov/2020/about/foundation-health-measures/Disparities.

[44] Guadamuz J.S., Kapoor K., Lazo M., Eleazar A., Yahya T., Kanaya A.M., Cainzos-Achirica M., Bilal U. (2021). Understanding Immigration as a Social Determinant of Health: Cardiovascular Disease in Hispanics/Latinos and South Asians in the United States. Curr. Atheroscler. Rep..

[45] Williams D.R., Selina A.M. (2013). Racism and health I: Pathways and scientific evidence. Am. Behav. Sci..

[46] In U.S., 14% With Likely COVID-19 to Avoid Care Due to Cost. https://news.gallup.com/poll/309224/avoid-care-likely-covid-due-cost.aspx.

[47] Vargas E.D., Juárez M., Sanchez G.R., Livaudais M. (2019). Latinos’ connections to immigrants: How knowing a deportee impacts Latino health. J. Ethn. Migr. Stud..

[48] Gramlich J. (2020). How Border Apprehensions, ICE Arrests and Deportations have Changed under Trump.

[49] Gómez-Aguiñaga B. (2016). Stepping into the Vacuum: State and Cities Act. on Immigration, but Do Restrictions Work?.

[50] Pedraza F.I., Nichols V.C., Lebrón A.M.W. (2017). Cautious Citizenship: The Deterring Effect of Immigration Issue Salience on Health Care Use and Bureaucratic Interactions among Latino US Citizens. J. Health Pol. Policy Law.

[51] Vargas E.D., Benitez V.L. (2019). Latino parents’ links to deportees are associated with developmental disorders in their children. J. Community Psychol..

[52] Ross E.J., Merton R.K. (1958). Social Theory and Social Structure. Am. Cathol. Sociol. Rev..

[53] Pearlin L.I., Schieman S., Fazio E.M., Meersman S.C. (2005). Stress, Health, and the Life Course: Some Conceptual Perspectives. J. Health Soc. Behav..

[54] Fothergill A., Maestas E.G.M., Darlington J.D. (1999). Race, Ethnicity and Disasters in the United States: A Review of the Literature. Disasters.

[55] Noh S., Kaspar V., Wickrama K.A.S. (2007). Overt and Subtle Racial Discrimination and Mental Health: Preliminary Findings for Korean Immigrants. Am. J. Public Health.

[56] Bailey Z.D., Krieger N., Agénor M., Graves J., Linos N., Bassett M.T. (2017). Structural racism and health inequities in the USA: Evidence and interventions. Lancet.

[57] Williams Institute (2013). LGBT Latino/a Individuals and Latino/a Same-Sex Couples. https://williamsinstitute.law.ucla.edu/publications/lgbt-latinx-indv-and-ss-couples/.

[58] CDC (Centers for Disease Control and Prevention) COVID Data Tracker 2021. https://www.cdc.gov/nchs/nvss/vsrr/covid19/index.htm..

[59] Joshi A., Kale S., Chandel S., Pal D.K. (2015). Likert Scale: Explored and Explained. Br. J. Appl. Sci. Technol..

[60] López N., Gadsden V.L., University of Pennsylvania, University of New Mexico (2016). Health Inequities, Social Determinants, and Intersectionality. NAM Perspect..

[61] López N. (2019). Chapter Contextualizing Lived Race-Gender and the Racialized-Gendered Social Determinants of Health. Mapping Race.

[62] Perreira K.M., Pedroza J.M. (2019). Policies of Exclusion: Implications for the Health of Immigrants and Their Children. Annu. Rev. Public Health.

[63] Pinedo M. (2020). The impact of deportation policies on the substance using behaviors of US-citizen Latinos. Int. J. Drug Policy.

[64] Frey W.H. (2018). Diversity Explosion: How New Racial Demographics Are Remaking America.

[65] Crenshaw K. (1991). Mapping the Margins: Intersectionality, Identity Politics, and Violence against Women of Color. Stanf. Law Rev..

[66] Evans C.R., Williams D.R., Onnela J.-P., Subramanian S. (2018). A multilevel approach to modeling health inequalities at the intersection of multiple social identities. Soc. Sci. Med..

[67] Perez-Truglia R. (2009). Applied Econometrics Using Stata.

[68] Long J.S., Freese J. (2006). Regression Models for Categorical Dependent Variables using Stata.

